# The Mouth Is the *Via Natura* for the Entrance of Disease

**Published:** 1897-11

**Authors:** M. V. Ball


### "THE MOUTH IS THE *VIA NATURA* FOR THE ENTRANCE OF DISEASE."[1]

BY M. V. BALL, M.D.

Mr. Chairman and Ladies and Gentlemen,—The mouth is the *via natura* for the entrance of disease.

When we examine the contents of our handkerchiefs we will find that a considerable amount of foreign material has been ensnared, so to speak, in the meshes of the lining membrane of the nose, and so prevented from gaining access to the lungs.

But in the mouth no such arrangement exists. We swallow the

---

[1] Read at a meeting of the Odontological Society of Pennsylvania, March 13, 1897.

air with our food, with the water we drink, and every time we use our vocal organs.

But not only from the air is disease—microbic disease—contracted. In the water and in the food itself many forms of infectious organisms reside, and under proper conditions develop disease.

Again, the teeth, liable to indentation and decay from use or neglect, are in such a state, like so many little test-tubes of microbe cultures, ready to inoculate the human body whenever and wherever its natural resistance is lowered. Even when the teeth are sound in every respect the spaces between the teeth become stored with food-remnants, which are liable to undergo putrefaction and act as a source of infection.

Bacteria are, as a rule, not very particular as to the source or quality of their nourishment. A minute quantity of organic material and a little moisture is, for the majority of micro-organisms, a suitable culture medium.

The refinements of the laboratory media are for other purposes than mere growth. For instance, gelatin media are useful because of the transparency and because they are readily converted into a fluid or solid state as desired. And so with agar, which is used because it remains solid at a higher temperature than gelatin, and can be used in the incubator for days without liquefying.

Only a few of the more important organisms require any particular form of food or special degree of temperature, and even these can develop, and do develop, under simpler conditions.

The natural secretions of the body, as the urine, milk, blood, and saliva, are very good germ foods, and have been artificially employed as such. Thus, one can understand why the mouth should be a favorable nidus for the culture of germs, and why so many varieties have been found therein.

The father of microscopy, and one might say of bacteriology,—namely, Antoon van Leeuwenhoek,—in 1683 contributed a paper to the Royal Society of London, describing a form of micro-organism that he discovered in the scrapings from between the teeth. We can readily understand why all of the varieties of bacteria commonly found in the air, food, and drink should be found in the mouth. And so various observers since Leeuwenhoek's time have isolated organisms from this location and given them special names.

Miller, whose work is classic, has cultivated twenty-five varieties. Biondi in 1887 described five species of pathogenic bacteria in the saliva alone. But besides the ordinary bacteria which accidentally gain entrance to the mouth, a few varieties have been discovered which seem to be normally present and have their residence in and about the teeth. These have been considered as the active agents in producing dental caries and other diseases of the teeth. Recognizing the fact that bacteria of a disease-producing nature are constantly present in the mouth, it is easy to understand that when the mouth is injured systemic infection readily occurs.

Thus, the eruption of teeth in an infant is very often accompanied by severe general disturbances,—meningitis, pneumonia, convulsions, diarrhœa, and simple fever. While many writers are disposed to speak lightly of the process of teething, my own experience—not very large, it is true—agrees with that of many older writers, that the complications occurring during teething are serious and directly due to the eruption. The gums are sensitive, are inflamed, and in condition to allow the bacteria ordinarily present in the mouth to gain access to the finer capillaries. A general infection results, and we have, perhaps, only a mild form of septicæmia,—fever, diarrhœa, etc.,—or we have a specific disease, like tubercular meningitis or pneumonia. The bacteria of pneumonia are very common residents of the mouths of otherwise healthy individuals. In uncleanly people and those who fondle the child by kissing the contagion is easily conveyed.

The eruptions on the skin, the nasal and aural catarrhs, and the stomach troubles which occur during dentition can all be considered as symptoms of one disease,—septicæmia.

We are frequently called to treat abscesses in the gums and cheeks, apparently arising from carious teeth. The teeth themselves are not at fault; it is the wound or injury made in the gum by a sharp piece of food, or perhaps inflammation started by the irritation of putrefactive material, that allows the pus-germs to make their way into the tissues. Here, finding a suitable soil, they speedily develop, forming gaseous abscesses, and, consequent thereon, general constitutional disturbances.

There are a number of local diseases of the mouth dependent largely on disease-germs. Stomatitis, while somewhat dependent on bacteria for its production, is, however, often caused by other agents, and helps to produce a bacterial infection.

Thrush, the first disease found to originate from a vegetable parasite,—first described in this light by Berg, of Stockholm, in 1846,—is not a rare occupant of the mouth.

Glanders is caused by a bacillus,—bacillus mallei.

Leprosy is caused by the lepra bacillus.

Actinomycosis, by the fungus of that disease,—actinomyces.

Gonorrhœa of the mouth has been noted.

Tubercular lesions of the mouth, strange to say, rarely occur. The sputum and bronchial discharge, charged with tubercle bacilli, while known to affect the stomach mucous membrane and very often the larynx, does not act on the mucous membrane of the mouth.

The sputum has been known to be germicidal for some species. Diphtheria bacilli, for instance, are destroyed in forty-eight hours by sputum, but pneumonia bacteria thrive in it.

There are a number of diseases of the teeth themselves which are supposed to lead to serious trouble in the maxillary bones; as, for instance, various inflammations of the pulp and cement. A number of investigators seem to find specific germs which cause dental caries.

As early as 1867 Leber and Rottenstein made public the theory that leptothrix penetrated the dental canal, dilating it, and in connection with acid destroyed the substance. It was thought that the sound enamel could be entered by the bacteria, but Miller proved that this was impossible and that the germ first produced an acid, which, by softening the tissue at some point, allowed the bacteria to enter. Ordinary mouth-fermentations produce sufficient lactic acid to act on the teeth. This fermentation itself is the result of acid-producing bacteria which are constantly present in the mouth. There is no organism that can be said to produce dental caries. Any acid-forming germ—and there are many varieties—can give the first injury, and once the enamel is attacked, caries is certain to follow. The affected tissue then becomes filled with cultures of bacteria, and six species have been isolated from the dentine by Gallipe and Vignal.

It seems more reasonable to suppose that dental caries, like decay of many other tissues, can occur under the influence of pus-forming organisms of various kinds.

The ordinary pus-germs are constantly present in the air, and therefore found very often in the mouth. Miller found the sputum of one hundred and eleven students fatal to mice in one hundred and one cases, and septicæmia was produced in nearly all the animals.

The dental caries in itself serves as a focus of disease. Glandular enlargement, especially of the neighboring lymphatic glands, is usually present. The wound, for such it really is, is in constant danger of infection by any of the more dangerous organisms that accidentally find lodgement in the mouth. It is not unlikely that diseases like acute rheumatism, endocarditis, meningitis, and even tuberculosis might arise through this entry. By rubbing vigorously a culture of the staphylococcus pyogenes aureus into his healthy arm one experimenter caused a number of boils to appear.

A local tuberculosis has resulted on the fingers of persons handling consumptives, when such fingers were injured. The ordinary scrofulitic swellings of the cervical glands which are so common in children are to-day believed to be tubercular,—local tuberculosis. Caries of the teeth, allowing the tubercular bacillus of the streets and of the food to gain entrance into the exposed lymph-channels, can thus cause the infection of neighboring glands.

Acute rheumatism, while not known to be dependent on a specific organism, is, however, so intimately associated with endocarditis, which itself is so often caused by the pneumonia bacteria, that it is not too far out of the way to consider rheumatism as probably of microbic origin.

There are probably predisposing causes, but the entrance of germs through the defective teeth is not to be overlooked. I have mentioned dentition as a condition during which the infant is peculiarly susceptible to microbic diseases. Convulsions are probably due to a sudden intoxication by the product of some virulent organism, the onset and termination are so much like an acute toxæmia.

Although in typhoid fever the tongue and teeth are very much affected by the accumulations on them, commonly known as sordes, yet the abscesses which often arise during convalescence originate probably through the intestinal lesions, and not from the mouth.

Of course, any foreign agency which injures the membrane—as, for instance, plates, artificial teeth, and so forth—establishes the same conditions for the entrance of disease as before considered. And the extraction of teeth is a surgical operation open to all the dangers of infection as much as any other operation. Until the wound made is healed, it seems to me, it should receive the same careful consideration as any other wound artificially produced in the mouth. Not only is it an open wound, but it is a wound continually exposed to the worst sort of infection. Unfortunately, I have not gone into this subject as studiously or carefully as it merits, but I trust that some thought may be aroused by the few facts here cited which will induce discussion and further research.

